# Marine drugs for cancer: surfacing biotechnological innovations from the oceans

**DOI:** 10.6061/clinics/2018/e482s

**Published:** 2018-08-03

**Authors:** Paula Christine Jimenez, Diego Veras Wilke, Leticia Veras Costa-Lotufo

**Affiliations:** I Departamento de Ciencias do Mar, Universidade Federal de Sao Paulo, Santos, SP, BR; II Nucleo de Pesquisa e Desenvolvimento de Medicamentos (NPDM), Departamento de Fisiologia e Farmacologia, Faculdade de Medicina, Universidade Federal do Ceara, Fortaleza, CE, BR; III Departamento de Farmacologia, Instituto de Ciencias Biomedicas, Universidade de Sao Paulo, Sao Paulo, SP, BR

**Keywords:** Anticancer Drug Development, Marine Biotechnology, Antibody-Drug Conjugates, Antimetabolites Drugs, Anti-tubulin Drugs, DNA-Alkylating Agents

## Abstract

This review will discuss the contributions of marine natural molecules, a source only recently found to have pharmaceutical prospects, to the development of anticancer drugs. Of the seven clinically utilized compounds with a marine origin, four are used for the treatment of cancer. The development of these drugs has afforded valuable knowledge and crucial insights to meet the most common challenges in this endeavor, such as toxicity and supply. In this context, the development of these compounds will be discussed herein to illustrate, with successful examples provided by cytarabine, trabectedin, eribulin and brentuximab vedotin, the steps involved in this process as well as the scientific advances and technological innovation potential associated with developing a new drug from marine resources.

## INTRODUCTION

Cancer remains one of the major issues for public health systems worldwide, and projections of the US National Cancer Institute have indicated an increase of 50% in cancer cases, reaching 21 million new cases in the next two decades 1. According to these projections, 7 out of 10 deaths caused by cancer will occur in Africa, Asia and Central and South America 2, challenging countries such as Brazil to improve strategies for surveillance, early diagnosis and effective treatment of cancer patients.

The usual forms of cancer treatment include surgery, radiotherapy and chemotherapy 3. Unlike surgery and radiotherapy, which are methods primarily indicated for solid tumors, chemotherapy is a form of systemic drug-based treatment that interferes with the process of growth and cell division in tumor cells. Currently, cancer chemotherapy is facing a remarkable revolution with the introduction of an increasing number of target-oriented drugs, increasing treatment efficacy, reducing side effects and improving the quality of life of patients 4.

However, despite the considerable arsenal of drugs available for cancer chemotherapy and the therapeutic success of various treatment regimens, existing therapies do not always achieve the expected results, as tumor recurrence and the onset of metastasis often occur 5,6. Therefore, the search for more selective compounds with fewer side effects, greater therapeutic potency and a lower resistance index for cancer therapy is of paramount importance.

### Natural products in cancer treatment

Natural products have made the greatest contribution to the development of drugs in cancer chemotherapy as the origin of over 70% of compounds in clinical use 7-9. Various examples of antineoplastic analogs obtained from plants and widely used therapeutically include vinblastine (Velban^®^), vincristine (Oncovin^®^) and their analogs vindesine (Eldisine^®^) and vinorelbine (Navelbine^®^); paclitaxel (Taxol^®^) and the analogs docetaxel (Taxotere^®^) and cabazitaxel (Jevtana^®^); podophyllotoxin and analogs etoposide (Etopophos^®^), teniposide (Vumon^®^) and belotecan (Camptobell^®^); and camptothecin and analogs topotecan (Hycamtin^®^) and irinotecan (Camptosar^®^). Soil bacteria are also a notable source of anticancer drugs, which can be clearly illustrated by compounds such as the anthracyclines doxorubicin (Doxil^®^; Adriamycin^®^), daunorubicin (Cerubidine^®^) and epirubicin (Ellence^®^); the glycopeptide bleomycin (Blenoxane^®^); and the non-ribosomal peptide dactinomycin (Cosmegen^®^) 9-11.

The compact and peculiar arrangement of natural molecules contains structural requirements that allow binding to specific targets or molecular interactions, resulting in phenotypic changes in biological systems 12-14. These properties are closely related to their pharmacological potential and therapeutic success in the most diverse diseases. There are estimates that 64% of all drugs currently registered have a natural product involved in their development 15,16.

There is extraordinary potential for the discovery of new naturally occurring anticancer drugs due to their invaluable biological diversity. To date, it has been estimated that less than 2% of plants have been analyzed for antineoplastic constituents, and even then, only cytotoxic activity was sought. In fact, a large number of molecules with antineoplastic activity derived from marine organisms, microorganisms and plants can still be revealed, and in addition, many known substances show unprecedented activities when tested against new therapeutic targets 10,17.

### Innovation from the sea

Seven marine-based pharmaceuticals have been approved for marketing, 23 compounds are in clinical trials between phases I and III, and over one thousand compounds isolated from marine organisms are undergoing preclinical studies 18-21. It should be noted that among those marine organism-derived compounds in clinical use, four are used in the treatment of cancer: cytarabine (Cytosar^®^), trabectedin (Yondelis^®^), eribulin mesylate (Halaven^®^) and the conjugated antibody brentuximab vedotin (Acentris^®^). Herein, we will discuss relevant elements of the four approved anticancer drugs, emphasizing the knowledge to overcome obstacles that make the process of natural product drug research and development an expensive, laborious and time-consuming endeavor.

### Cytarabine (Cytosar) – Debuting marine compounds in the cancer field

**Figure 1:**
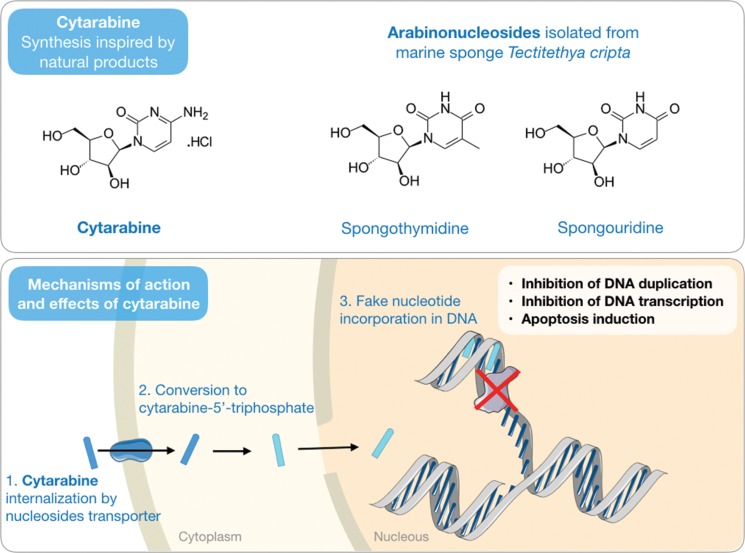
Chemical structures of cytarabine and related natural products, spongothymidine and spongouridine, isolated from the marine sponge *Tectitethya cripta* (top). A schematic representation of the mechanism of action of cytarabine in cancer cells (bottom).

Werner Bergmann from Yale University and a group of collaborators throughout the 1940s and 1950s published several articles in a series they called “contributions to the study of marine products” 22-26. The most significant of these works was the isolation of the arabinonucleosides spongothymidine and spongouridine (Figure 1) from the Caribbean sponge *Cryptotethya crypta* (now renamed *Tectitethya crypta*) 23. The discovery of these nucleosides was further entwined with two important scientific developments: the first was breaking the existing paradigm that a nucleoside would have biological function only if it had ribose or deoxyribose in its structure; and the second was the introduction of the pharmacological concept of antimetabolites in the context of anticancer chemotherapy, with the synthesis of analogs of natural arabinonucleosides and cytosine arabinose and the development of cytarabine (or Ara-C).

An antimetabolite drug is one that has a structure sufficiently similar to that of a metabolite produced naturally by the body such that it could be recognized but lacks the ability to preserve its function, thereby interfering with normal cell metabolism 27. In this context, similar to that of its natural prototypes, the mechanism of action of cytarabine is based on its rapid conversion to the respective triphosphate arabinonucleoside through sequential phosphorylation. The triphosphocytarabine then becomes a DNA polymerase substrate and is subsequently incorporated into the DNA in place of a cytosine. However, due to the presence of arabinose in place of deoxyribose, the cytarabine triphosphate, once bound to DNA, prevents the formation of a phosphodiester bond between the two pentoses and, therefore, the elongation of the DNA strain, thus interrupting the process of DNA synthesis 28,29 (Figure 1).

Cytarabine was approved for clinical use in 1969 and is still widely used in the treatment of various types of leukemia, such as acute lymphocytic leukemia, acute myelogenous leukemia, chronic myelogenous leukemia, and non-Hodgkin's lymphoma. Although this molecule is a synthetic analog and not the natural product itself, cytarabine is historically reported as the first example of a commercially available marine-derived drug.

However, it is important to emphasize that the principle of using a nonfunctional nucleoside to inhibit the elongation of the DNA strand, which was first observed with the arabinonucleosides of *C. crypta*, is a strategy used in other chemotherapeutic treatments. Another of these analogs, adenine arabinose, has been developed into the antiviral drug vidarabine (or Ara-A), which has been clinically used since 1976 for the treatment of *Herpes simplex* and *Varicella zoster*, among other viral infections. Furthermore, the antiviral azidothymidine (AZT or zidovudine), the first available treatment for AIDS and part of the WHO model list of essential drugs, deserves attention. This molecule, a thymidine analog in which the pentose is linked to an N chain, was synthesized in 1964 with anticancer claims that it did not fulfill. However, when the spread of AIDS rapidly turned into an epidemic in the mid-1980s, in the search for an anti-HIV compound, AZT proved to be extremely effective in blocking the viral reverse transcriptase enzyme, which is crucial for HIV retrovirus replication 30.

### Trabectedin (Yondelis^®^) – Overcoming the supply problem

**Figure 2:**
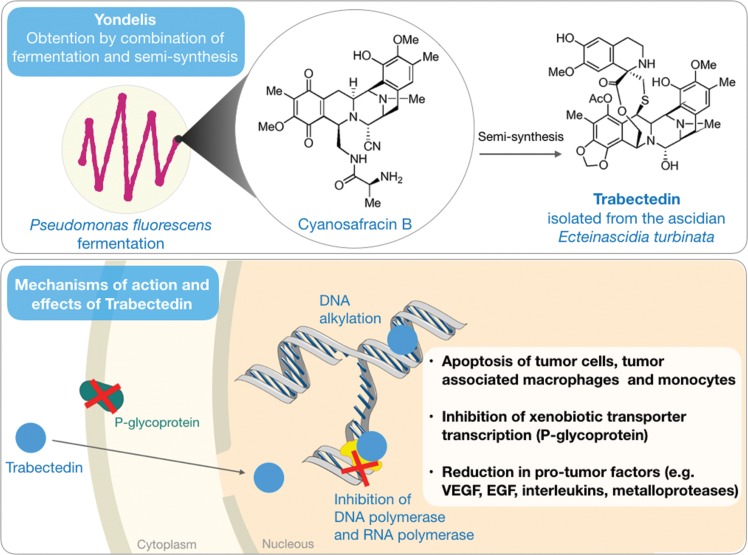
Chemical structures of trabectedin isolated from the ascidian *Ecteinascidia turbinata* and its precursor cyanosafracin B isolated from the bacteria *Pseudomonas fluorescens* (top). A schematic representation of the mechanism of action of trabectedin in cancer cells (bottom).

The anticancer activity of extracts obtained from the Caribbean ascidian *Ecteinascidia turbinata* has been known since the 1960s 31, but the isolation and structural elucidation of its active principle occurred more than 20 years later, as described by Rinehart et al. 32 and Wright et al. 33. ET-743, also called ecteinascidin 743 or trabectedin (Figure 2), was identified as the most abundant of the six structurally related alkaloids produced by ascidian.

Mode of action studies recognized this molecule as a DNA alkylator with a mechanism that differs from that of most typical alkylating agents (Figure 2). Trabectedin binds to guanine residues arranged in specific sequences in the minor groves of the double-helix, further bending DNA strands toward the opposite side of the alkylation site 34,35. These adducts created by trabectedin prevent DNA transcription by arresting RNA polymerase II activity 36-38. Inhibition of MDR1 gene transcription is one of the most relevant effects, as this gene is responsible for the production of P-glycoprotein, which participates in the cellular detoxification process 39,40. Additionally, the transcription and consequent production of chaperones, such as heat shock proteins (HSPs), are also affected 41,42. The TC-NER pathway (repair by nucleotide excision coupled to transcription), which is recruited after RNA polymerase II arrest to repair DNA lesions detected during the transcription process, also appears to be compromised by trabectedin. Moreover, it has been shown that TC-NER-deficient cells are resistant to the effect of this compound 35,36.

Also worthy of mention are the recent findings on the effects of trabectedin on the tumor microenvironment (TME). The TME contains nontumor cells, such as macrophages, which actively communicate with tumor cells. These macrophages release inflammatory mediators as well as growth and angiogenic factors that enable the proliferation and metastasis of tumor cells. While TME cells are normally quiescent and have low sensitivity to most chemotherapy treatments, trabectedin has been shown to reduce the tumor inflammatory response in addition to activating caspase 8 and selectively inducing apoptosis in monocytes and macrophages 43.

Trabectedin was obtained by total synthesis for the first time in 1996 using an extremely complex process with low yield of the final product 44. Years later, the same group was able to simplify the process and improve compound yield; however, the chemical synthesis did not prove to be effective in providing trabectedin in sufficient quantities to meet the demands of the clinic. Cultivation of the ascidian in marine farms (or mariculture) was also considered an option to address issues related to the supply of the molecule. However, the production of trabectedin in cultivated *Ecteinascidia turbinata* was inconsistent and generally reduced when compared to that in organisms found under natural ecological circumstances, as 1 ton of cultivated ascidian was sufficient for the isolation of merely 1 g of the molecule 45. The supply of trabectedin to ensure commercialization of the product and its clinical use resulted in the development of a semisynthetic process from the intermediate cyanosafracin B, a large-scale antibiotic obtained from the bacterium *Pseudomonas fluorescens* 46.

Trabectedin, under the trademark Yondelis^®^, was developed by PharmaMar, a pharmaceutical company dedicated exclusively to research and development of drugs from marine organisms. Notably, this was the first marine natural molecule approved for clinical use in cancer chemotherapy and was the first drug in 20 years to be validated for the treatment of soft tissue sarcoma. Yondelis^®^ received authorization from the European Medicines Agency (EMEA) for marketing in 2007. The FDA, however, approved the use of Yondelis^®^ only in 2015.

### Eribulin Mesylate (Halaven) – Understanding structure-activity relationships

**Figure 3:**
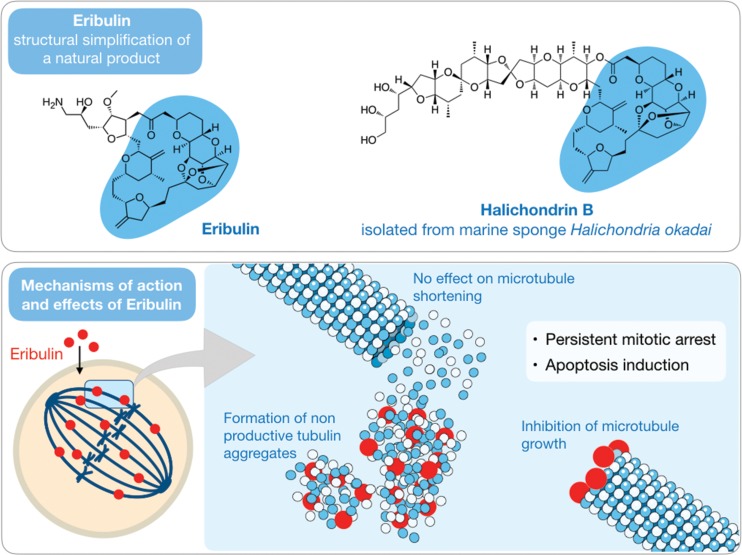
Chemical structures of eribulin mesylate and the related natural product halichondrin B isolated from the sponge *Halichondria okadai* (top). A schematic representation of the mechanism of action of eribulin mesylate in cancer cells (bottom).

Halichondrins are a series of macrocyclic polyethers with pronounced anticancer activity both *in vitro* and *in vivo* that were isolated by Hirata and Uemura 47 from the sponge *Halichondria okadai* collected on the coast of Japan at the Miura Peninsula. Among them, halichondrin B (Figure 3), a compound with high molecular weight (approximately 1,110 g/mol), served as a prototype for eribulin, an analog in the form of a mesylate salt (Figure 3).

The route of total synthesis of halichondrin B was determined by Aicher et al. 48. In addition, studies of the relationship between structure and biological activity demonstrated that it was the macrocyclic ring and not the side chain that afforded anticancer activity to this molecule 49. Thus, eribulin mesylate, which has maintained almost only the macrocyclic ring, nearly halving its molecular weight (to 729.90 g/mol), is to date the most complex drug obtained by total synthesis 49.

Eribulin mesylate, a derivative of the natural molecule, is a potent antimitotic compound that acts as an inhibitor of microtubule dynamics with an *in vitro* IC_50_ in the low nM range. The specific target of this compound is tubulin, whereby binding to specific regions prevent polymerization and, consequently, arrest the extension of microtubules (Figure 3). By these means, the cell remains in an irreversible mitosis blockage, and the prolongation of this state finally leads to death by apoptosis 50. Eribulin was developed by Eisai, a Japanese-based pharmaceutical company that has a research operation in the USA, with considerable support from the National Cancer Institute (NCI) during this process. In 2010, Halaven^®^, or eribulin mesylate, was approved by the FDA to treat advanced metastatic breast cancer, and in 2016, it became the second line of treatment for liposarcoma therapy 21.

### Brentuximab vedotin (Adcetris) – Targeted cytotoxic drugs

In 1972, extracts obtained from the gastropod mollusk *Dolabella auricularia*, which is found in the Indian Ocean, stood out for their pronounced anticancer activity. Nevertheless, only 15 years later, the peptides called dolastatins were identified as the active compounds of these extracts, among which dolastatins 10 and 15 exhibited potent antiproliferative activities against several tumor cell lines 51. Tubulin was also established as the main target of action of these peptides; therefore, the blockade of microtubule polymerization and, consequently, cell division is the mechanism by which dolastatins inhibit the proliferation of tumor cells 52.

**Figure 4:**
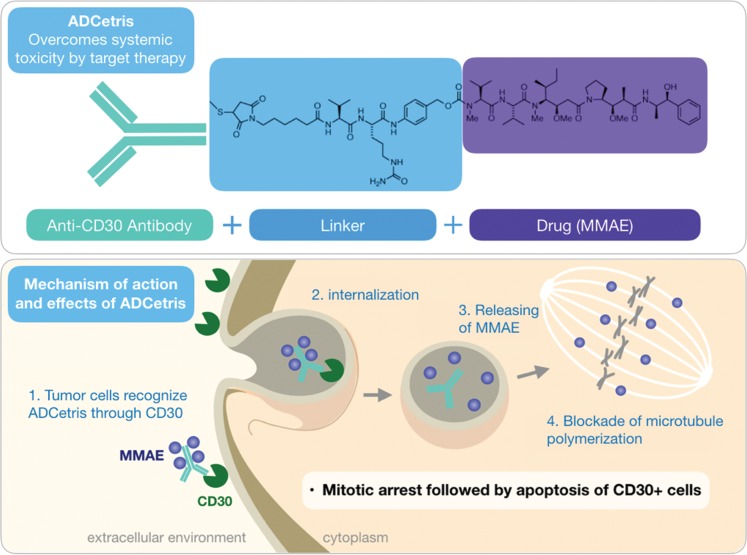
Structure of brentuximab vedotin, an antibody-drug conjugate developed with the active principle monomethylauristatin E, a derivative of the natural product dolastatin 10 isolated from the mollusk *Dolabella auricularia* (top). A schematic representation of the mechanism of action of brentuximab vedotin in cancer cells expressing the CD30 antigen on the membrane (bottom).

Despite the extraordinary *in vitro* activity of dolastatins – dolastatin 10 demonstrated IC_50_ in the order of pM against a panel of tumor cells – none of these peptides, nor their synthetic derivatives, such as auristatin (soblidotin), cematodine, and syntatodine, progressed beyond phase II in clinical trials 53,54. However, monomethylauristatin E (MMAE or vedotin), a derivative of auristatin, successfully became the warhead of the antibody-drug complex (ADC) brentuximab vedotin (Figure 4) developed by the US-based company Seattle Genetics and known commercially as Adcetris® 55,56. ADCs are composed of a monoclonal antibody covalently linked to the pharmacological agent 57. In the specific case of brentuximab vedotin, the antibody is directed against CD30, a membrane protein recognized as a tumor marker for some types of lymphomas. Thus, vedotin selectively targets cells expressing CD30, and some types of tumor cells do so in greater amounts. Recognition of the brentuximab antibody by the CD30 protein leads to the internalization of the ADC, and within the cell, proteolytic cleavage releases vedotin from the complex. In turn, free vedotin reaches its target of action, blocking cell division and, ultimately, inducing cell death 56 (Figure 4).

Adcetris^®^ was approved in 2012 for the treatment of Hodgkin's lymphoma, a type of cancer that is distinguished by the high expression of CD30. MMAE has also been included in the formation of other ADCs that are currently undergoing clinical trials, with the antibody of the complex directed to different membrane proteins. Glembatumumab vedotin (CDX-011) recognizes cells with NBM transmembrane glycoprotein expression and is being tested in patients with advanced melanoma as well as those with metastatic breast cancer. Pinatuzumab vedotin and polatuzumab vedotin, which are antibodies directed at CD22 and CD79b proteins, respectively, are being tested for the treatment of leukemias and lymphomas 58.

In Brazil, the bioprospection of marine organisms with biomedical properties is rapidly expanding but falls short when considering the richness of the biodiversity among the diverse ecosystems of coast and islands. During the last decade, substantial effects of this expansion can be confirmed, such as increased scientific production, structuring of research networks, training of human resources and deposits of patents. Our research group has focused on the isolation of biologically relevant molecules from invertebrates from the Brazilian coast for the past 17 years 59-62. Lately, to pursue sustainability and broaden the bioprospective regions, the group has begun to examine marine bacteria, including both free-living and invertebrate endophytes, for their anticancer potential 63-67. Microorganism collection and isolation efforts from diverse matrixes and localities, ranging from the coast to remote islands, have led to a current in-house bank of over 1,000 bacterial strains. This collection has been screened for cytotoxicity against tumor cells, and some strains have been further evaluated in other contexts, either chemical or biological, using the traditional phenotypic approach or a target-oriented strategy.

Nevertheless, ventures in this area are essentially academic and, consistent with the bioprospection of medicinal plants, are considerably more widespread in the country; there is a dissonance with the industrial sector, thus allowing the underutilization of the biological resource. Nevertheless, Brazil has been identified by Conservation International as a megadiverse country, a title backed by the large share of biological diversity housed within our territorial boundaries that is shared with only 16 other nations. In this sense, these yet subproductive salty waters that bathe our coast are expected to shelter quite a generous pharmacological potential.

## AUTHOR CONTRIBUTIONS

Jimenez PC, Wilke DV and Costa-Lotufo LV contributed equally to the writing and reviewing of the manuscript and approved the final version.
